# Myocarditis Revealing a Pathogenic Desmoplakin (DSP) Variant: Implications for Genetic Testing Pathways

**DOI:** 10.7759/cureus.116569

**Published:** 2026-09-20

**Authors:** Blend Reka, Jason N Dungu

**Affiliations:** 1 School of Medicine, Anglia Ruskin University, Chelmsford, GBR; 2 Essex Cardiothoracic Centre, Basildon and Thurrock University Hospitals NHS Foundation Trust, Basildon, GBR

**Keywords:** arrhythmogenic cardiomyopathy, desmoplakin cardiomyopathy, dsp mutation, genetic testing, myocarditis

## Abstract

Desmoplakin (DSP) variants are associated with arrhythmogenic cardiomyopathy and can present with myocarditis-like “hot phases” before overt structural disease develops. We report a 30-year-old woman who presented with chest tightness, palpitations, and elevated troponin and was diagnosed with acute myocarditis on cardiac magnetic resonance imaging. In view of a family history of myocarditis in her younger sister, genomic testing was pursued following multidisciplinary review, despite the absence of a dedicated myocarditis testing pathway. A pathogenic heterozygous DSP splice-site variant (c.939+1G>A) was identified and was also detected in her asymptomatic father, who had a normal cardiac phenotype. Over the following 16 months, the patient experienced eight further episodes of myocarditis-like symptoms, resulting in nine episodes in total. These were characterized by recurrent chest pain and mild troponin elevation with minimal or no elevation of inflammatory markers, while biventricular systolic function remained preserved. This case illustrates recurrent myocarditis as a potential early manifestation of DSP-related cardiomyopathy, including in the absence of global systolic dysfunction. It also highlights the diagnostic value of genomic testing in patients with recurrent myocarditis, particularly in the presence of a relevant family history. Identification of a pathogenic DSP variant can inform longitudinal surveillance, prognostic assessment, and cascade screening of relatives and supports consideration of clearer genomic testing pathways for selected patients presenting with recurrent myocarditis.

## Introduction

Myocarditis is an inflammatory disease of the myocardium that can present with acute chest pain. Its etiology is broad, encompassing viral infections, autoimmune conditions, and increasingly recognized genetic predispositions [1]. While most cases of myocarditis follow a self-limiting course, a subset presents with recurrent episodes, and a family history of myocarditis raises the possibility of an underlying genetic cardiomyopathy.

Desmoplakin (DSP) cardiomyopathy is a form of arrhythmogenic cardiomyopathy that primarily involves the left ventricle, with occasional right ventricular involvement [2]. It is characterized by ventricular wall inflammation and fibrosis, causing episodes of acute myocardial injury and promoting ventricular arrhythmias [3]. The DSP gene encodes a desmosomal protein critical for cardiomyocyte adhesion [2]. Essentially, desmosomes hold heart muscle cells together mechanically.

A distinctive feature of DSP cardiomyopathy is the occurrence of "hot phases," which are discrete episodes of acute chest pain, troponin elevation, and electrocardiographic changes in the absence of obstructive coronary artery disease, closely mimicking acute myocarditis and frequently preceding the development of overt structural disease [4,5]. DSP variants are the most commonly implicated genetic variants in such hot-phase presentations, accounting for 69% of cases in a systematic review [4].

Despite the growing body of evidence, myocarditis is not currently an indication for genetic testing within the National Genomic Testing Directory (NGTD) in England, and no dedicated myocarditis gene subpanel exists. This stands in contrast to the 2025 European Society of Cardiology (ESC) guidelines on myocarditis and pericarditis, which recommend genetic testing in patients with recurrent myocarditis, a relevant family history, significant ventricular arrhythmias, or a heavy scar burden [1]. This diagnostic gap risks delaying diagnoses that carry significant implications for prognosis, arrhythmic risk stratification, and family screening.

We present the case of a 30-year-old woman with nine episodes of myocarditis-like presentations over 16 months, in whom a pathogenic DSP variant was ultimately identified following multidisciplinary team (MDT) review. This case illustrates the clinical consequences of the current genomic testing gap and supports the consideration of a dedicated myocarditis gene subpanel within the NGTD.

## Case presentation

A 30-year-old woman with no prior cardiac history presented to the emergency department on Day 1 with chest pain, chest tightness, dyspnea, dizziness, palpitations, and five episodes of syncope, each lasting approximately 30 seconds. Her only significant background was a family history of myocarditis in her younger sister, with whom she was estranged.

On initial assessment, a 12-lead electrocardiogram (ECG) demonstrated ST elevation in leads II, III, and aVF only, with a concave morphology, but was not diagnostic for pericarditis in isolation (Figure 1). Blood tests revealed a significantly elevated high-sensitivity troponin of 201-212 ng/L (upper limit of normal <14 ng/L) and a mildly elevated C-reactive protein (CRP) of 8 mg/L (normal <5 mg/L). N-terminal pro-B-type natriuretic peptide (NT-proBNP) was within the normal range at 69 ng/L (Table 1). Viral serology was negative for cytomegalovirus (CMV), Epstein-Barr virus (EBV), and coxsackievirus, making an infective etiology less likely. Transthoracic echocardiography demonstrated normal cardiac structure and preserved left ventricular systolic function with a left ventricular ejection fraction (LVEF) of 60%.

**Figure 1:**
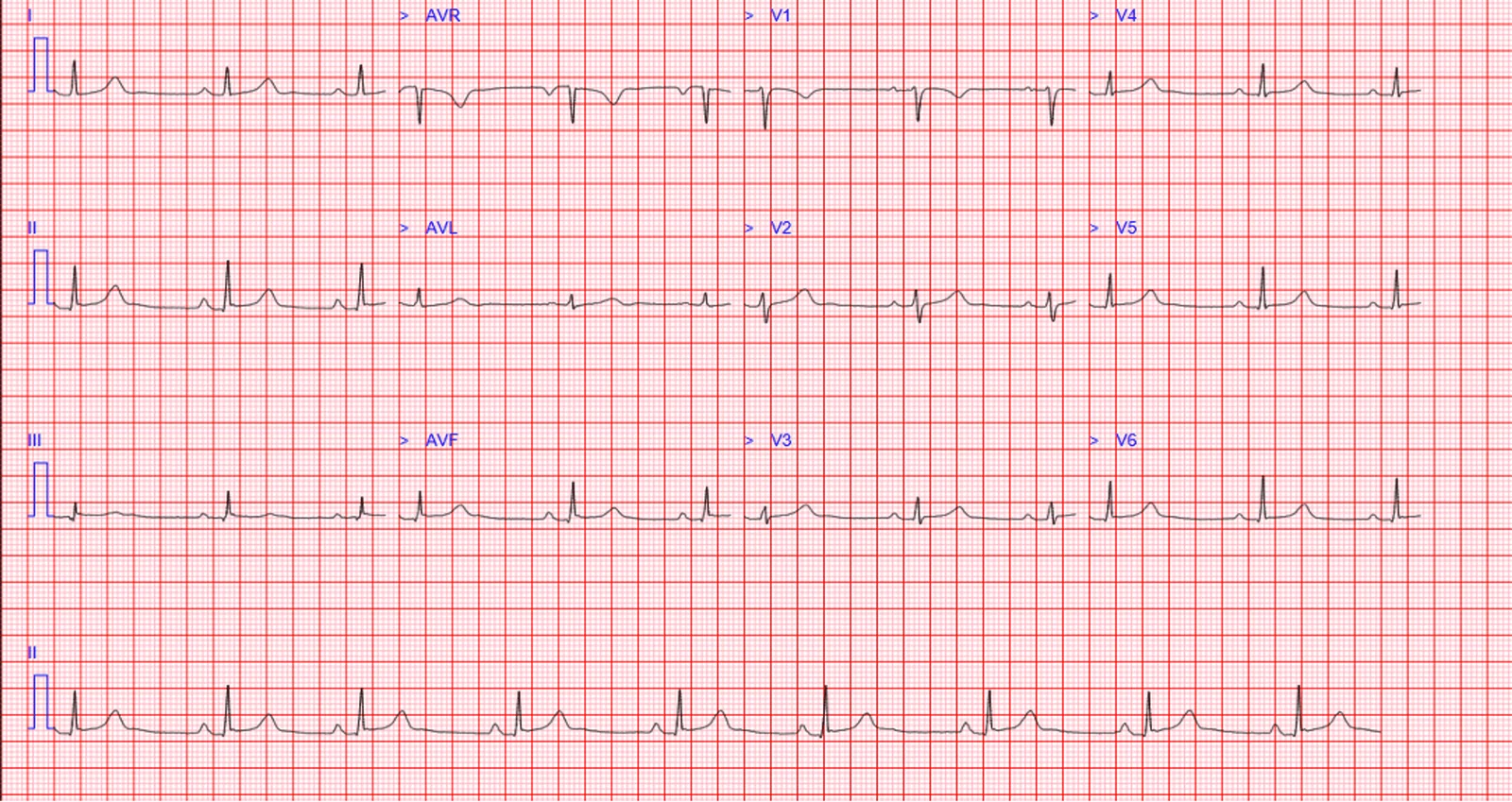
12-lead ECG ECG showing ST elevation in leads II, III, and aVF. ECG, electrocardiogram

**Table 1:** Initial laboratory investigation findings

Parameters	Patient values	Reference range
High-sensitivity troponin	201-212 ng/L	<14 ng/L
C-reactive protein (CRP)	8 mg/L	<5 mg/L
N-terminal pro-B-type natriuretic peptide (NT-proBNP)	69 ng/L	<125 ng/L
Cytomegalovirus (CMV) serology	Negative	Negative
Epstein-Barr virus (EBV) serology	Negative	Negative
Coxsackievirus serology	Negative	Negative

CT coronary angiography (CTCA) demonstrated unobstructed coronary arteries, excluding an ischemic etiology. Cardiac magnetic resonance imaging (CMRI) demonstrated normal left and right ventricular size and systolic function, with an LVEF of 67% and a right ventricular ejection fraction of 57%. A bulge at the apical cavity of the right ventricle was noted but not deemed diagnostic for an overt cardiomyopathy. There was subepicardial late gadolinium enhancement (LGE) at the basal inferolateral segments with corresponding myocardial edema on T2-weighted sequences, consistent with acute myocarditis (Figures 2, 3). However, given the significant family history and minor right ventricular morphological changes, arrhythmogenic ventricular cardiomyopathy with acute inflammatory changes was considered in the differential diagnosis. The patient was treated for acute myopericarditis with colchicine, nonsteroidal anti-inflammatory drugs (NSAIDs), bisoprolol, and lansoprazole for gastroprotection.

**Figure 2:**
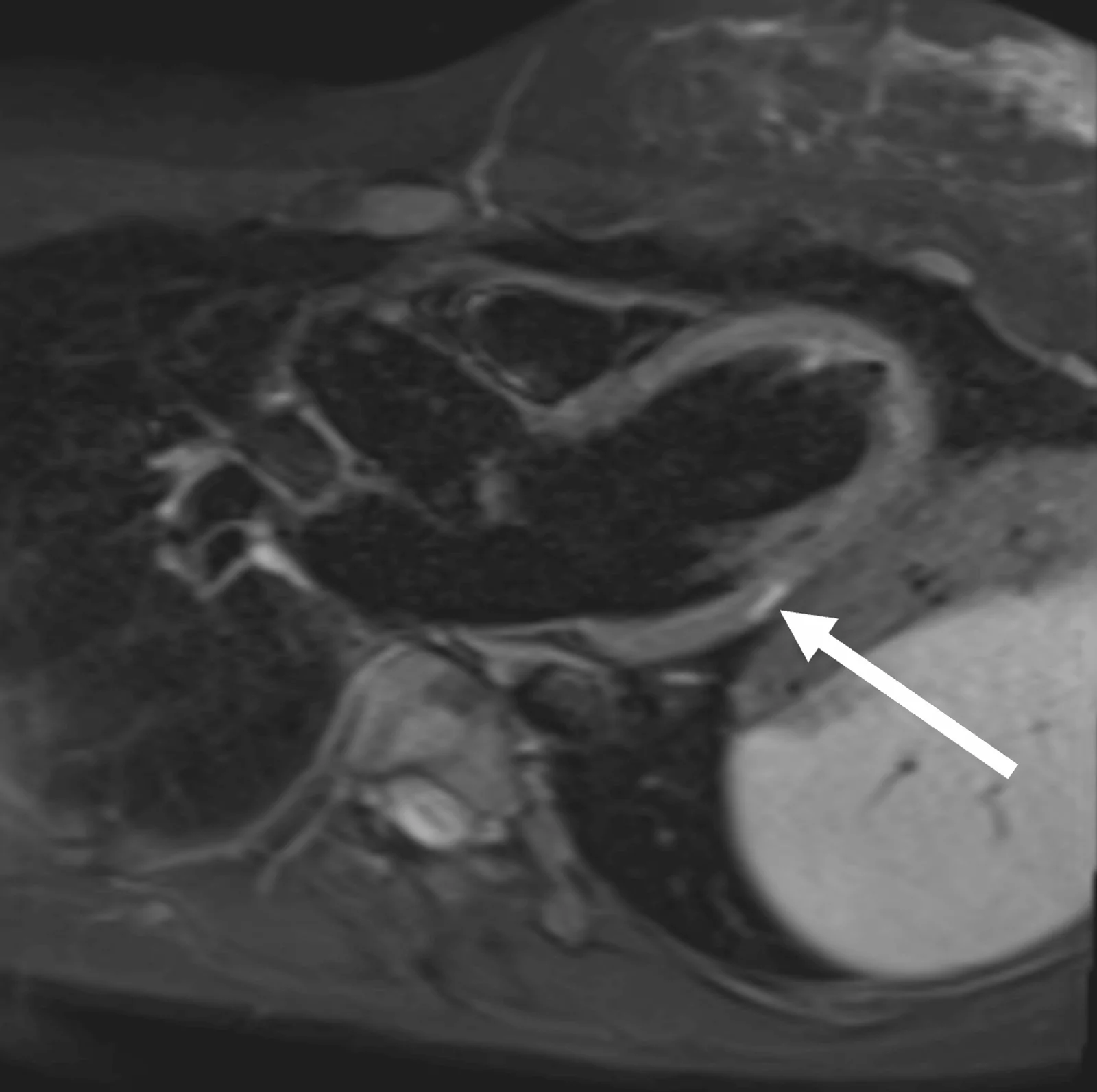
Cardiac MRI T2 STIR Arrow showing edema in the mid-inferolateral wall. STIR, short tau inversion recovery

**Figure 3:**
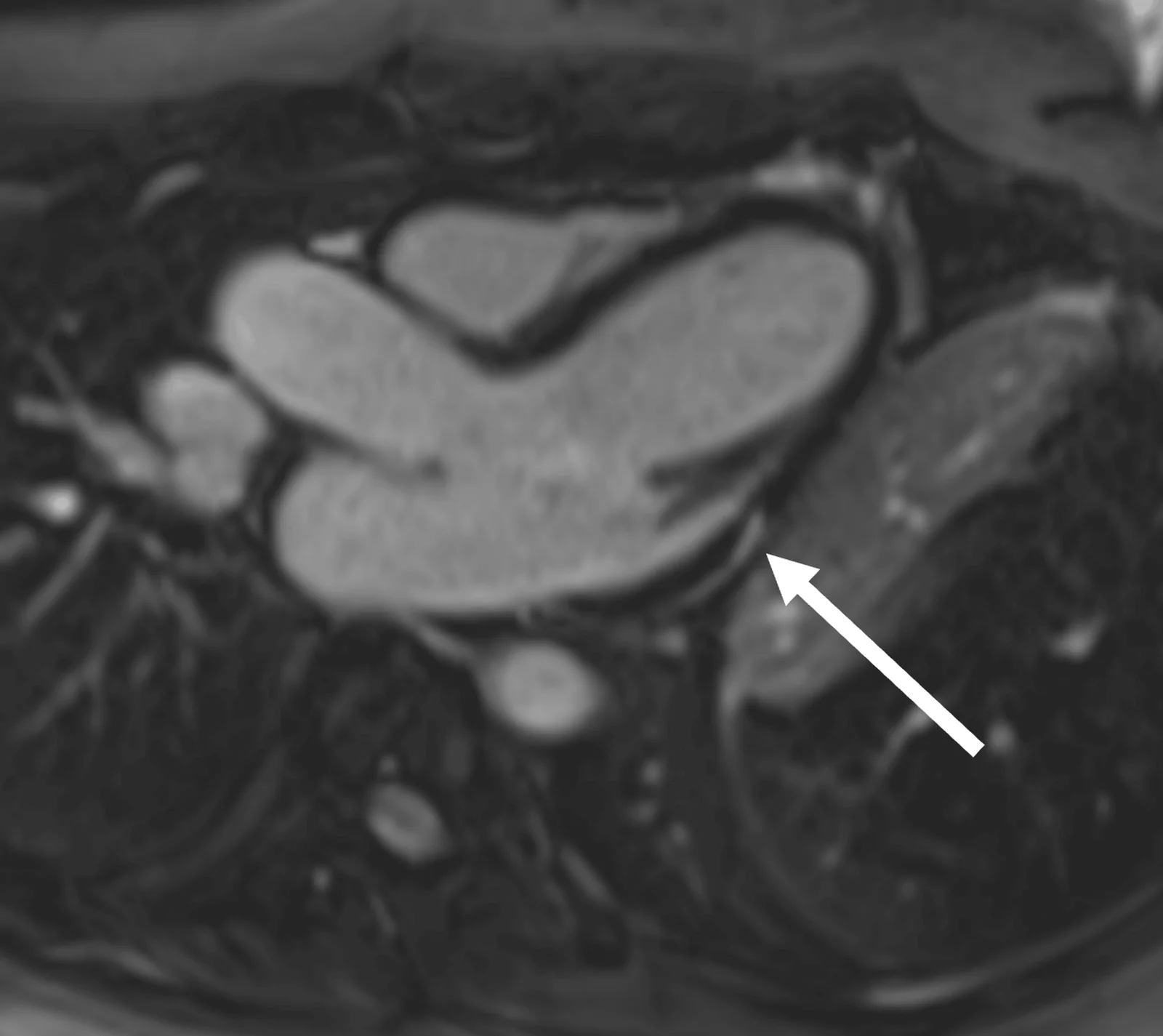
Cardiac MRI showing late gadolinium enhancement Arrow showing subepicardial enhancement in the basal-to-mid inferolateral wall.

Between approximately Month 3 and Month 16, she experienced eight further episodes of myocarditis-like presentation, bringing the total to nine episodes over 16 months. Each episode was characterized by recurrent chest pain radiating to the back and dizziness, with a mild troponin elevation of 105-126 ng/L. Notably, CRP remained consistently normal or minimally elevated throughout (1-9 mg/L), indicating recurrent myocardial injury in the absence of significant systemic inflammation. Biventricular function remained preserved across all episodes.

The combination of recurrent myocarditis, a positive family history, and the absence of an infective or autoimmune trigger raised strong suspicion for an underlying genetic cardiomyopathy. An initial request for genetic testing was declined, as myocarditis is not a primary indication for genomic testing and no dedicated myocarditis gene subpanel exists within the NGTD. Following further MDT discussion, genetic testing was subsequently approved and undertaken; this identified a pathogenic splice-site variant in the DSP gene (c.939+1G>A) in the heterozygous state, confirming a diagnosis of DSP cardiomyopathy associated with arrhythmogenic cardiomyopathy. Parental cascade screening identified the same pathogenic variant in her father, who remained entirely asymptomatic with a normal cardiac phenotype.

At the time of most recent review, the patient reported ongoing chest pain radiating to the back, worsened by inspiration and minimal exertion, alongside dyspnea and lethargy on minimal activity. Given the persistence of symptoms despite conservative management and following MDT review, prednisolone 30 mg daily was commenced for four weeks with a planned weaning schedule of 5 mg decrements. Initiation of azathioprine was recommended following further MDT discussion due to recurrent symptoms after initial prednisolone weaning and as a steroid-sparing agent. However, following discussion of the proposed treatment, the patient declined azathioprine, and an alternative agent, hydroxychloroquine, was initiated; the response will be assessed during follow-up in the Cardiology and Rheumatology clinics. Long-term colchicine has been continued, as a prior attempt to discontinue it was associated with a subjective flare of myocarditis. Additionally, the patient will be serially assessed for implantable cardioverter-defibrillator (ICD) implantation, given the potential risk of ventricular arrhythmias in the context of confirmed DSP cardiomyopathy and right ventricular abnormality; the decision will be guided by the detection of any ventricular arrhythmias on implantable loop recordings or if there is any decline in left ventricular systolic function.

## Discussion

The presentation of this patient, characterized by recurrent episodes of myocarditis, electrocardiographic features of pericarditis, cardiac magnetic resonance (CMR) findings consistent with acute myocarditis, and a positive family history, is in keeping with the recognized inflammatory or "hot-phase" phenotype of DSP cardiomyopathy [2,4,5]. The association between pathogenic DSP variants and arrhythmogenic cardiomyopathy was first described by Rampazzo et al. in 2002, who identified a DSP variant segregating with arrhythmogenic cardiomyopathy in an Italian family [6]. Since then, accumulating evidence has established a strong association between DSP variants and adverse cardiac outcomes, including ventricular arrhythmias and myocarditis-like presentations that may mimic acute myocardial infarction, with chest pain, ST-segment elevation, and unobstructed coronary arteries [4,7].

Recognition of patients who may have an underlying inherited cardiomyopathy is essential because the acute presentation of DSP cardiomyopathy frequently mimics isolated acute myocarditis. This case highlights an important gap between contemporary clinical guidelines and current genomic testing provision in England. The 2025 ESC Guidelines for the Management of Myocarditis and Pericarditis recommend consideration of genetic testing in patients with recurrent myocarditis, a relevant family history, significant ventricular arrhythmias, or extensive myocardial scar burden [1]. Our patient fulfilled multiple of these criteria, including recurrent myocarditis and a first-degree relative with myocarditis, yet the initial request for genetic testing was declined because myocarditis is not currently recognized as an indication within the NGTD, and no dedicated myocarditis gene subpanel exists. This case is not unique in demonstrating such a diagnostic delay; Fatrous et al. similarly described a prolonged diagnostic pathway in a patient with DSP cardiomyopathy presenting as recurrent myocarditis [8]. However, our case further highlights the clinical consequences of this gap and supports consideration of a dedicated myocarditis gene subpanel within the NGTD, incorporating genes such as DSP, FLNC, PLN, and LMNA, which are increasingly recognized as causes of inflammatory cardiomyopathy phenotypes [4,5].

A notable feature of this case was the consistent dissociation between troponin and CRP across all nine episodes. While each episode showed a troponin elevation of 105-212 ng/L, CRP remained normal or only minimally elevated throughout, never exceeding 8 mg/L at the index presentation and falling to 3 mg/L in all subsequent episodes. This pattern suggests that myocardial injury in DSP cardiomyopathy may be driven primarily by mechanical disruption of desmosomal integrity rather than classical inflammatory pathways [3]. This distinction mattered clinically, as the MDT was initially unable to justify starting immunosuppression in the absence of objectively raised inflammatory markers. This case therefore highlights the need for DSP-specific diagnostic and management thresholds, rather than relying on inflammatory myocarditis guidelines that were not developed with specific genotypes in mind.

Management of recurrent inflammatory episodes in DSP cardiomyopathy remains challenging, as there is no specific literature on the management of this condition. Initial management with colchicine, NSAIDs, and beta-blockers was in keeping with conventional acute myopericarditis management. Long-term colchicine was subsequently continued given the documented flare of symptoms following its discontinuation. Immunosuppression with corticosteroids was initially withheld given the absence of significant CRP elevation and the transient nature of the troponin rise. The evolution in management with prednisolone and subsequent switch to a steroid-sparing regimen was based on anecdotal evidence and reflects the genuine clinical uncertainty that exists in this condition and the absence of clear guidelines for escalation of immunosuppression in DSP-related recurrent myocarditis [7,9].

Given the evidence and risk of ventricular arrhythmias in DSP cardiomyopathy, use of an ICD for primary prevention is being considered in this case and has also been widely recommended in these patients, as highlighted in a recently reported case of DSP CM presenting with recurrent myocarditis episodes [8]. Early genetic testing may facilitate prompt specialist assessment and consideration of primary prevention ICD therapy before the occurrence of potentially life-threatening arrhythmias, although this was not indicated in this case. Our patient had a first-degree relative with myocarditis, which ultimately strengthened the indication for genetic testing; however, a relevant family history is not always present. Reliance on family history alone therefore risks delaying the diagnosis of inherited cardiomyopathies in patients presenting with recurrent myocarditis. This highlights the need for greater clinical awareness of inherited inflammatory cardiomyopathies and supports broader consideration of genetic testing in patients with recurrent myocarditis or other high-risk features, even in the absence of an apparent family history, in keeping with contemporary ESC recommendations [1].

This case is subject to the limitations of a single case report, and the findings cannot be generalized to all patients with isolated or recurrent myocarditis episodes. Management decisions in this case were guided by MDT consensus in the absence of validated management guidelines, and longer-term follow-up will be required to determine the impact of these interventions on disease progression and arrhythmic outcomes.

## Conclusions

This case illustrates that DSP cardiomyopathy should be considered in patients presenting with recurrent myocarditis, particularly in the presence of a relevant family history and in the absence of significant systemic inflammatory markers. The diagnostic delay experienced by this patient underscores the need for a dedicated myocarditis gene subpanel within the NGTD. Early genetic diagnosis carries important implications for arrhythmic risk stratification, family screening, and long-term surveillance.
